# The association between dog walking, physical activity and owner’s perceptions of safety: cross-sectional evidence from the US and Australia

**DOI:** 10.1186/s12889-016-3659-8

**Published:** 2016-09-22

**Authors:** Hayley Christian, Lisa Wood, Andrea Nathan, Ichiro Kawachi, Stephen Houghton, Karen Martin, Sandra McCune

**Affiliations:** 1 School of Population Health, The University of Western Australia, 35 Stirling Highway, Crawley, WA 6009 Australia; 2 Telethon Kids Institute, 100 Roberts Road, Subiaco, WA 6008 Australia; 3 Institute for Health & Ageing, Australian Catholic University, Level 6, 215 Spring St, Melbourne, VIC 3000 Australia; 4 School of Public Health, Harvard University, 677 Huntington Avenue, Kresge Building 7th Floor, Boston, Massachusetts 02115 USA; 5 Centre for Child and Adolescent Related Disorders, The University of Western Australia, 35 Stirling Highway, Crawley, WA 6009 Australia; 6 Waltham® Centre for Pet Nutrition, Freeby Lane, Waltham-on-the-Wolds, Melton Mowbray, Leicestershire LE14 4RT UK

**Keywords:** Dog, Physical activity, Walking, International, Neighborhood, Safety, Community

## Abstract

**Background:**

We examined the relationship between dog walking and physical activity within and between four US cities and Australia and investigated if dog walking is associated with higher perceived safety in US and Australian cities.

**Methods:**

Dog owners (*n* = 1113) in the Pet Connections Study completed a cross-sectional survey. Data were collected across four study sites; three in the US (San Diego, Nashville, Portland) and a fourth in Australia (Perth). Physical activity, local walking, dog walking, and individual and community perceptions of safety were analysed for dog walkers and non-dog walkers for each study site. Between-city comparisons were examined for dog walkers.

**Results:**

Across all study sites, dog walkers walked with their dog 5–6 times/week for a total of 93–109 min/week and achieved ≥30mins of physical activity on more days/week and walked in their neighbourhood more often/week, compared with non-dog walkers (all *p* ≤ 0.01). Compared with Perth, significantly fewer dog walkers walked in their local park in the three US study sites. San Diego dog walkers walked more often in their neighborhood/week compared with Perth dog walkers (all *p* ≤ 0.05).

In Portland, dog walkers perceived significantly more neighborhood problems and in Nashville dog walkers perceived a significantly higher level of neighborhood natural surveillance (i.e., ‘eyes on the street’), compared with non-dog walkers (both *p* ≤ 0.05). Among dog walkers, females were more likely than males to feel safer walking with their dog in their neighborhood (OR = 2.49; 95 % CI = 1.76, 3.53). Compared with dog walkers in Perth, dog walkers from each of the US study sites felt safer in their neighborhood and perceived there was more neighborhood surveillance (all *p* ≤ 0.001).

**Conclusion:**

This multi-site international study provides further support for the potential for dog walking to increase levels of daily physical activity. Walking with a dog may be a mechanism for increasing perceptions of neighborhood safety and getting to know the neighborhood, however significant between-country differences exist. Further international research is required to understand the drivers for these between-country differences. Community based programs and policies aimed at improving safety and social connectedness should consider the wider community benefits of dog walking and include strategies for supporting more dog walking.

**Electronic supplementary material:**

The online version of this article (doi:10.1186/s12889-016-3659-8) contains supplementary material, which is available to authorized users.

## Background

Globally, physical inactivity is a significant health issue. Physical inactivity is the fourth leading risk factor for mortality behind high blood pressure, tobacco use and high blood glucose and a significant factor in rising obesity rates [1]. Cost-effective, sustainable community based strategies for increasing physical activity levels are required. A key determinant and strategy for increasing physical activity is the social support for walking provided by family and friends [2–6]. An often over-looked source of social support for walking is the family dog. Walking with a dog has been shown to be an important source of motivation and social support for dog owners encouraging them to regularly walk [7–9].

A large proportion of the community own dogs [10]. In the US and Australia approximately 40 % of households own one or more dogs [11], while in the UK and Europe the level of dog ownership is lower (25 %) [10]. Dog ownership, in particular dog walking, is a significant correlate of physical activity and of meeting the recommended level of physical activity required for health benefits [12]. In a review of the evidence approximately 60 % of dog owners walked their dog for an average of 160 min per week and four walks per week [12]. Importantly, this review made a number of recommendations including the need for international studies of the human-health benefits of dog walking [12].

One of the main mechanisms through which dogs facilitate increased physical activity is through the social support provided whilst dog walking [7, 8, 13]. However, dog walking also provides a number of other community level social benefits [9, 14, 15]. Dog walking may contribute to increased owner and community perceptions of safety. Qualitative research shows that owners (particularly women) feel safer when walking with their dog and suggests that dog ownership and dog walking may be a deterrent for local crime [7, 9, 16, 17]. For example, dog walkers who are regularly out and about in the neighborhood walking with their dog may be an important source of ‘eyes on the street’. This type of natural surveillance provides opportunities for people to monitor their neighborhood, which is associated with increased feelings of safety [18]. Moreover, greater natural surveillance is associated with reduced physical disorder (e.g., vandalism) and graffiti [19]. However, dog walking as a potential source of natural surveillance has not been empirically tested. Thus, considering a large proportion of the community own a dog and on average 60 % of owners walk their dog, investigation of the community and individual level safety benefits of dog walking is warranted.

The aim of the study was: 1) to examine the relationship between dog walking and physical activity within and between four cities across the US and Australia; and 2) to investigate if dog walking is associated with greater perceived safety in those cities. We hypothesised that the relationship between dog walking and physical activity would be consistent across cities; that dog owners who are out and about in their local neighborhood walking with their dog have more positive perceptions of their local neighborhood; and that they perceive there to be higher levels of natural surveillance (‘eyes on the street’), fewer neighborhood problems and feel safer.

## Methods

### Sample and procedure

The sample included all dog owners (*n* = 1113; 41.3 %) participating in the Pet Connections Study. Pet Connections is a cross-sectional study designed to examine the relationship between pet ownership and social capital, including between and within country (US vs. Australia) differences, and to identify pet-related factors that precipitate and maintain social connectedness in neighborhoods. The methodology is reported elsewhere [14, 20], and is briefly described here. To be eligible, participants were required to be aged ≥ 18 years and to have lived in their neighborhood for at least one year. Overall, 2692 adults participated in a cross-sectional telephone survey across four study sites (San Diego, US *n* = 690, Response Rate (RR) = 45.8 %; Nashville, US *n* = 664, RR = 44.1 %; Portland, US *n* = 634, RR = 42.1 %; Perth, Australia *n* = 704; RR = 60.2 %), yielding an overall response rate of 47.3 %. Study site samples were representative of the wider population in terms of sex, age group, neighborhood socio-economic status and pet ownership rates [20]. The survey was administered to coincide with the autumn and early winter months in the US (September to December 2012) and Australia (April to June 2012).

### Socio-demographic measures

Socio-demographic variables included: age, gender, highest education level attained (secondary or less, vocational training, bachelor degree or higher or other), ethnicity (US only – White/Caucasian, Hispanic or Latino Descent, Black/African American, Asian and Other), country of birth (Australia only – Australia or Overseas), number of dependents <18 living at home, number of years lived in neighborhood and type of residence (house, duplex, townhouse or villa, apartment or flat or other). Participants were also asked whether they owned a dog [21].

### Physical activity measures

Number of days per week participants reported ≥30 min of moderate-vigorous physical activity were recorded [22]. Participants reported the frequency and location (e.g., park, streets, beach/river) of where they walked or jogged in their neighbourhood in a usual week. These items were based upon the Neighborhood Physical Activity Questionnaire (NPAQ), which has acceptable reliability [23]. Items from the Dogs and Physical Activity (DAPA) tool were used to collect frequency and duration of dog walking per usual week [21]. These items have excellent test-rest reliability (frequency of dog walking/week intra-class correlation (ICC) = 0.98; duration of dog walking/week ICC = 0.94) [21]. Dog owners who reported that they walk or jog with their dog (s) were classified as ‘dog walkers’.

### Perceptions of safety and the neighborhood

General neighbourhood perceptions of safety were based on existing scales. The ‘Feel Safe In Neighborhood’ scale was based on four items (i.e., Feel safe: walking alone in daytime in neighborhood; walking alone at night in neighborhood; using parks in neighborhood; and in own home) (Cronbach’s α = 0.78) [24, 25]. The ‘Neighborhood Natural Surveillance’ scale included three items (i.e., see people out walking and jogging; people in neighborhood feel it is a safe place to live; and few people walk down my street) (Cronbach’s α = 0.50) [14, 24]. Participants also reported whether their perception of neighborhood safety was enhanced by three items relating to natural surveillance (i.e., I feel safer when I see people walking; I feel safer when I see people out walking with their dog; and I feel less safe when I see deserted streets and parks) (Cronbach’s α = 0.60) [24]. All items in scales were measured using a 4-point Likert scale (1 = strongly disagree; 4 = strongly agree). Items in each scale were summed and then averaged.

The perceived potential ‘Neighborhood Problems’ scale measured graffiti and/or vandalism, crime, traffic, neighborhood maintenance (6 items: inadequate lighting at night, houses or yards not well looked after, poor upkeep of parks and public open space, trash or litter in public areas, vacant or run-down buildings, and poor street lighting) and social incivilities (3 items: noisy neighbours or loud parties, drug dealing or drug use, people not cleaning up after dogs) [24–28]. All items in this scale were measured using a 4-point Likert scale (1 = strongly disagree; 4 = strongly agree). Items were dichotomised (agree vs. disagree) and summed (range 0–12) (Cronbach’s α = 0.89). Details of this measure are reported elsewhere [29].

Two items measured neighborhood perceptions specifically in relation to dog walking. Owners reported whether being with a dog helped them to feel safer when out walking or jogging and whether walking or jogging with their dog enabled them to get to know their neighborhood (dichotomous: true/false) [14].

### Statistical analyses

Analyses were restricted to dog owners only (*N* = 1113). Chi-square tests were used to examine socio-demographic differences between dog walkers and non-dog walkers for each study site and overall. Linear regression was used to examine the relationship between dog walking status (independent variable) and days/week of ≥30mins of moderate-vigorous physical activity and frequency of walking in the neighbourhood/week (dependent variables). Logistic regression was used to examine the relationship between dog walking status (independent variable) and if participants walked to their local park in a usual week (dependent variable). Two models were run for each physical activity-related dependent variable; unadjusted and then adjusted for age group, sex, highest education level, ethnicity (US), country of birth (Australia), number of children in household, housing type, and time lived in neighborhood. Descriptive analyses (mean and standard error) were conducted for frequency and duration of dog walking/week. All analyses were conducted separately for each study site with non-dog walkers as the reference group. Between city comparisons were performed for dog walkers only (reference site = Perth), using the Bonferroni correction procedure for hypothesis testing involving multiple comparisons.

Linear regression was used to examine the relationship between dog walking status (independent variable) and perceptions of safety dependent variables (Feel safe in neighborhood; Neighborhood problems; Neighborhood surveillance; Feel safe if have neighborhood surveillance). All models were adjusted for age group, sex, highest education level, ethnicity (US), country of birth (Australia), number of children in household, housing type, and time lived in neighborhood. All analyses were conducted separately for each study site with non-dog walkers as the reference group. Between city comparisons were examined for dog walkers only (reference site = Perth) using Bonferroni correction of *p*-values for multiple comparisons.

Finally, descriptive analyses were conducted by study site and overall to examine the percentage of dog walkers who reported that they ‘Feel safer walking with dog’ and ‘Got to know neighborhood through walking dog’. Non-dog walkers were excluded from analyses as our analyses were focused on dog walking only. Logistic regression was used to examine the relationship between gender (independent variable; reference group = males) and the proportion of participants reporting they ‘Feel safer walking with dog’ and ‘Got to know neighborhood through walking dog’ (dependent variables). All models adjusted for age group, highest education level, ethnicity (US), country of birth (Australia), number of children in household, housing type, and time lived in neighborhood. All analyses were conducted separately for dog walkers from study site and overall. Between city comparisons were performed for a) all dog walkers (reference site = Perth) and b) female dog walkers only (reference site = Perth) correcting our *p*-values for multiple comparisons using the Bonferroni procedure.

## Results

Overall, 47 % of dog owners were male, 22 % were 40–49 years, 40 % had a bachelor degree or higher, 39 % had one or more children living at home and 56 % of dog owners were classified as ‘dog walkers’.

### Socio-demographic factors associated with dog walking status in the US and Australia

Overall, there were no significant associations between age group, sex, education level, ethnicity, country of birth, number of children living in household, time lived in current neighbourhood or housing type by dog walking status (Table 1). The majority of US dog owners were White/Caucasian and the majority of Perth dog owners were born in Australia compared with overseas. There were no significant associations between any socio-demographic factors and dog walking status for Perth, San Diego and Portland. However in Nashville, a significantly higher proportion of dog walkers compared with non-dog walkers had a bachelor degree and lived in a townhouse or apartment (both *p* ≤ 0.05).

**Table 1:** Sample characteristics by dog walking status across study sites

	TOTAL (*n* = 1113)		San Diego (*n* = 276)		Portland (*n* = 233)		Nashville (*n* = 296)		Perth (*n* = 308)	
	Non-dog walker^1^ (*n* = 490) %	Dog walker (*n* = 623) %	Non-dog walker^1^ (*n* = 134) %	Dog walker (*n* = 142) %	Non-dog walker^1^ (*n* = 110) %	Dog walker (*n* = 123) %	Non-dog walker^1^ (*n* = 141) %	Dog walker (*n* = 155) %	Non-dog walker^1^ (*n* = 105) %	Dog walker (*n* = 203) %
Age group^2^	*p* = 0.124		*p* = 0.194		*p* = 0.127		*p* = 0.166		*p* = 0.508	
18–29 years	17.6	17.0	29.9	26.8	10.9	8.9	9.9	15.5	19.0	16.3
30–39 years	18.4	21.3	24.6	25.4	20.9	31.7	20.6	25.2	4.8	9.4
40–49 years	24.7	20.2	13.4	16.2	33.6	23.6	34.0	23.2	17.1	18.7
50–59 years	17.1	22.0	12.7	20.4	15.5	18.7	14.2	18.7	28.6	27.6
60+ years	20.8	18.6	19.4	11.3	16.4	17.1	19.1	14.2	29.5	28.1
Male	*p* = 0.758		*p* = 0.381		*p* = 0.243		*p* = 0.177		*p* = 0.356	
	46.5	47.4	44.0	49.3	49.1	41.5	41.8	49.7	53.3	47.8
Highest education level^3^	*p* = 0.102		*p* = 0.182		*p* = 0.771		*p* = 0.046*		*p* = 0.607	
Secondary or less	35.1	29.9	33.6	25.4	26.4	29.3	36.2	21.3	44.8	39.9
Vocational training	22.0	25.7	26.9	28.2	21.8	26.8	18.4	21.3	21.0	26.6
Bachelor degree or higher	38.0	41.6	32.1	43.0	49.1	40.7	39.7	53.5	31.4	32.0
Other	2.9	1.8	3.0	2.1	1.8	2.4	4.3	2.6	1.9	0.5
Ethnicity (US only)^4^	*p* = 0.374		*p* = 0.087		*p* = 0.149		*p* = 0.674		-	-
White/Caucasion	70.6	75.2	49.3	64.1	88.2	86.2	77.3	76.8		
Hispanic or Latino Descent	14.5	9.5	35.8	21.8	1.8	4.9	4.3	1.9		
Black/African American	7.5	8.3	6.0	6.3	3.6	0.0	12.1	16.8		
Asian	2.3	2.3	4.5	2.1	0.9	2.4	1.4	1.3		
Other	3.4	3.4	3.0	4.9	3.6	2.4	3.5	1.9		
Country of birth (Australia only)^5^	-		-		-		-		*p* = 0.258	
Australia									70.5	64.0
Overseas									29.5	36.0
Number of children living in household^6^	*p* = 0.277		*p* = 0.466		*p* = 0.608		*p* = 0.387		*p* = 0.079	
None	59.0	61.2	55.2	55.6	53.6	61.8	61.0	62.6	66.7	63.5
One	14.9	17.0	14.9	21.1	15.5	9.8	19.1	18.7	8.6	17.2
Two	16.1	15.2	17.2	12.7	20.0	19.5	12.8	14.2	15.2	15.3
Three or more	9.2	6.1	11.9	10.6	9.1	8.1	7.1	3.2	8.6	3.9
Time lived in neighborhood	*p* = 0.262		*p* = 0.671		*p* = 0.642		*p* = 0.275		*p* = 0.084	
1–3 years	16.7	17.0	24.6	21.8	18.2	13.0	14.9	21.9	7.6	12.3
4–9 years	18.8	24.2	18.7	26.1	22.7	31.1	22.0	23.2	10.5	20.2
10–14 years	21.0	19.1	19.4	19.0	20.9	17.9	17.0	14.8	28.6	23.2
15–20 years	13.7	12.7	9.7	7.7	12.7	12.2	13.5	16.8	20.0	13.3
More than 20 years	29.8	27.0	27.6	25.4	25.5	26.8	32.6	23.2	33.3	31.0
Housing types^7^	*p* = 0.066		*p* = 0.407		*p* = 0.113		*p* = 0.007*		*p* = 0.422	
House	88.6	85.9	82.1	79.6	95.5	87.0	90.1	82.6	87.6	92.1
Duplex	4.3	2.7	2.2	4.9	2.7	1.6	6.4	2.6	5.7	2.0
Townhouse or villa	3.1	4.0	8.2	4.2	0.0	1.6	0.0	5.2	3.8	4.4
Apartment or flat	2.9	5.1	6.0	9.2	0.9	4.9	2.1	7.1	1.9	1.0
Other	1.2	1.4	1.5	1.4	0.9	3.3	1.4	1.3	1.0	0.5

*p ≤ 0.05; ^1^Reference group; Missing data: ^2^12; ^3^17; ^4^14; ^6^7; ^7^5; ^5^ For Australia country of birth measure typically used (rather than ethnicity)

### Physical activity behavior by dog walking status in the US and Australia

Across all study sites, on average dog walkers walked with their dog 5–6 times per week for a total of 93–109 min per week (Table 2). Dog walkers achieved ≥30 min of moderate-vigorous physical activity on more days per week and walked in their neighbourhood more often per week, compared with non-dog walkers (all *p* ≤ 0.05). These findings were consistent across all four study sites. Similarly, across all four study sites a greater proportion of dog walkers compared with non-dog walkers walked in their local park (all *p* ≤ 0.001). Compared with Perth, significantly fewer dog walkers walked in their local park in the three US study sites (80 % vs. 28–45 %; *p* ≤ 0.001). Moreover, San Diego dog walkers walked more often in their neighborhood each week (Mean 7.6; Standard Error (SE) 4.0) compared with Perth dog walkers (Mean 6.4; SE 3.8; *p* ≤ 0.05). Models remained significant after adjustment for socio-demographic factors (Additonal file 1: Table S1).

**Table 2:** Within and between city differences in physical activity behaviour by dog walking status

	San Diego (*n* = 276)		Portland (*n* = 233)		Nashville (*n* = 296)		Perth (*n* = 308)		Significant between city comparisons (Dog walkers)^2^
	Non-dog walker^1^ (*n* = 134) Mean (SE)	Dog walker (*n* = 142) Mean (SE)	Non-dog walker^1^ (*n* = 110) Mean (SE)	Dog walker (*n* = 123) Mean (SE)	Non-dog walker^1^ (*n* = 141) Mean (SE)	Dog walker (*n* = 155) Mean (SE)	Non-dog walker^1^ (*n* = 105) Mean (SE)	Dog walker (*n* = 203) Mean (SE)	*p*-value
≥30mins moderate-vigorous physical activity (days/week)	3.5 (2.5)	4.3 (2.2)**	3.2 (2.5)	4.0 (2.2)*	2.9 (2.3)	4.1 (2.2)***	2.8 (2.6)	3.9 (2.6)***	0.467
Frequency of neighbourhood walking/week	2.3 (2.7)	7.6 (4.0)***	2.1 (2.6)	6.5 (3.8)***	1.6 (2.1)	6.7 (4.4)***	1.9 (2.8)	6.4 (3.8)***	0.027 PE < SD
Walk in local park (%)	17.2	45.1***	12.7	42.3***	11.3	28.4***	19.0	80.3***	0.000 PE > SD, PL, NV
Frequency of dog walking/week	-	6.0 (4.0)	-	5.1 (3.6)	-	5.3 (3.9)	-	5.0 (3.4)	0.074
Minutes of dog walking/week	-	108.6 (134.8)	-	93.5 (106.3)	-	108.1 (142.9)	-	92.8 (115.0)	0.510

*p ≤ 0.05; **p ≤ 0.01; ***p ≤ 0.001; ^1^Reference group; *SD* San Diego, *PL* Portland, *NV* Nashville, *PE* Perth

^1^Reference group = Non-dog walker

^2^Reference group = Perth

### Neighborhood perceptions of safety by dog walking status

Some variation in general perceptions of the neighborhood by dog walking status was found across the four study sites. In Portland, dog walkers compared with non-dog walkers perceived significantly more neighborhood problems (β = 2.20; 95 % CI = 0.38, 4.02) (Table 3). Nashville dog walkers perceived a significantly higher level of neighborhood natural surveillance, compared with non-dog walkers (β = 0.44; 95 % CI = 0.08, 0.79). There were no within-site differences between dog walkers and non-dog walkers for perceived safety in the neighborhood or perceived safety provided by other people out walking (i.e., neighborhood surveillance).

**Table 3:** Within and between city differences in dog walkers and non-dog walkers perceptions of safety

	San Diego (*n* = 276) β (95 % CI)^1^	Portland (*n* = 233) β (95 % CI)^1^	Nashville (*n* = 296) β (95 % CI)^1^	Perth (*n* = 308) β (95 % CI)^1^	Significant between city comparisons (Dog walkers only)^2^ *p*-value
Feel safe in neighborhood^3^	−0.13 (−0.61,0.36)	−0.01 (−0.54,0.53)	0.27 (−0.22,0.76)	0.00 (−0.43,0.47)	0.000 PE < SD, PL, NV
Neighborhood problems^4^	0.98 (−0.61,2.56)	2.20 (0.38,4.02)*	0.51 (−1.07,2.09)	−0.29 (−1.62,1.04)	0.08 PE > NV*
Neighborhood natural surveillance^3^	0.21 (−0.16,0.57)	−0.88 (−0.45,0.28)	0.44 (0.08,0.79)*	0.29 (−0.04,0.62)	0.000 PE < PL, NV
Feel safe if have neighborhood natural surveillance^3^	0.07 (−0.29,0.43)	0.10 (−0.28,0.48)	0.20 (−0.17,0.57)	0.11 (−0.21,0.43)	0.000 PE < SD, PL, NV

*p ≤ 0.05; *SD* San Diego, *PL* Portland, *NV* Nashville, *PE* Perth

^1^All models adjusted for age group, sex, highest education level, ethnicity (US); country of birth (Aust), number of children in household, housing type, time livedin neighborhood; Reference group = Non-dog walker

^2^Reference group = Perth

^3^Measured on a 4-point Likert scale: 1 = strongly disagree; 4 = strongly agree

^4^Count of neighborhood problems (range 0–12)

Among dog walkers, those in the US study sites, compared with those in Perth, felt safer in their neighborhood, perceived that there was more neighborhood surveillance and perceived that they would feel safer if there were neighborhood surveillance (all *p* ≤ 0.001). Moreover, dog walkers in Nashville perceived there to be significantly fewer neighborhood problems compared with dog walkers in Perth (*p* ≤ 0.05).

### Gender differences in perceptions of safety when walking with dog and getting to know the neighborhood through dog walking

Dog walkers were asked about feelings of safety whilst dog walking and whether they had got to know their neighborhood through dog walking (Table 4). Overall and for each study city (except Nashville), females were more than twice as likely as males to feel safer walking with their dog in their neighborhood (Overall: OR = 2.49; 95 % CI = 1.76, 3.53). No statistically significant between city comparisons were observed. There were however, between city gender differences for getting to know the neighborhood through dog walking. Compared with Perth dog walkers, Nashville dog walkers were less likely to have got to know their neighborhood through walking their dog (*p* ≤ 0.05). When stratified by gender, female dog walkers in Portland compared with Perth were more likely to have got to know their neighborhood through walking their dog (*p* ≤ 0.05).

**Table 4:** Within and between city differences in dog walker’s neighborhood perceptions by gender

	All Dog walkers (*n* = 623)		San Diego Dog walkers (*n* = 142)		Portland Dog walkers (*n* = 123)		Nashville Dog walkers (*n* = 155)		Perth Dog walkers (*n* = 203)		Significant between city differences (All dog walkers)^2^	Significant between city differences (Female dog walkers)^3^
	Overall %	OR^1^ (95 % CI)	Overall %	OR^1^ (95 % CI)	Overall %	OR^1^ (95 % CI)	Overall %	OR^1^ (95 % CI)	Overall %	OR^1^ (95 % CI)	*p*-value	*p*-value
Feel safer walking with dog	58.6	**2.49 (1.76,3.53)**	57.7	**2.54 (1.09,5.93)**	58.5	**4.17 (1.57,11.09)**	58.7	0.97 (0.45,2.07)	59.1	**6.07 (2.99,12.33)**	0.971	0.277
Got to know neighborhood through walking dog	77.4	0.74 (0.49,1.11)	83.1	0.65 (0.20, 2.09)	81.3	1.60 (0.49,5.24)	71.0	0.69 (0.29,1.66)	75.9	0.50 (0.23,1.08)	**0.010** PE > NV	**0.024** PE < PL

Bolded text = *p* ≤ 0.05; *SD* San Diego, *PL* Portland, *NV* Nashville, *PE* Perth, Overall percentages are unadjusted

^1^Models examining gender differences adjusted for age group, highest education level, ethnicity (US); country of birth (Aust), number of children in household, housing type, time lived in neighborhood. Reference group = Males

^2^Reference group = Perth

^3^Between city differences for FEMALE dog walkers only; Reference group = Perth

## Discussion

A recent review of the evidence and meta-analysis called for international studies of the relationship between dog walking and physical activity [12]. Our study is one of the first international studies to investigate this relationship. Overall, our findings highlight that the proportion of dog owners who report walking their dog and the relationship between dog walking status and physical activity behavior is consistent both within and between developed countries such as the US and Australia.

Furthermore, the frequency and duration of dog walking was consistent between and within the two countries. Regardless of country or city of residence, dog walkers reported walking their dog 5–6 times per week (for approximately 93–109 min/week). In comparison, a review of the evidence reported a weekly median frequency and duration of dog walking of 4 walks and 160 min, respectively [12]. These values were based on all dog owners and the studies used varying measures of dog walking behavior. In contrast, the current study used the same consistent dog walking measure across all study sites and averaged dog walking frequency and mean minutes per week for those owners who did some walking with their dog.

International studies provide an opportunity to determine how robust the evidence base is across countries. They also allow a deeper understanding of the cultural and social and physical environment influences on the relationship between dog walking and physical activity. Differences between dog walkers and non-dog walkers were observed but were not consistent across all four study sites. Nashville dog walkers perceived a significantly higher level of neighborhood natural surveillance and Portland dog walkers perceived significantly more neighborhood problems, compared with non-dog walkers. It is possible that as a result of dog walkers being out in the community walking their dog they notice more about the neighborhood they live in and are able to more accurately recall the features of their neighborhoods whether they be positive or negative attributes. A similar relationship has been observed in studies of the influence of neighborhood perceptions on physical activity behavior. For example, physical activity levels were higher in people who perceived their neighborhoods as unclean and untidy compared with those who perceived a high level of neighborhood cleanliness [18, 30]. Our study observed that a high proportion of dog walkers (77 %) reported that they had got to know their neighborhood through walking their dog. Moreover, a greater proportion of dog walkers compared with non-dog walkers walked in their local park. It is possible that dog walkers that are out and about in their neighborhood, are more aware of their local neighborhood and that their perceptions of their neighborhood environment may more closely reflect the actual environment. Further research is required to determine the match (or mismatch) between perceptions and objective measures of the neighborhood environment for dog walkers and non-dog walkers.

Dog walking itself may provide an important source of ‘eyes on the street’. Dog owners who exercise their dog are out and about in their neighborhood on a near daily basis, as reflected in the mean frequency of dog walking per week (i.e., 5–6 times/week) found in this study and a review [12]. Dog walkers physical presence in the community may contribute to the collective safety of the community through being a source of ‘eyes on the street’. Pedestrians make streets safer, lively and interesting to watch which encourages surveillance and greater visibility in the public realm thus increasing people’s feelings of safety [18, 31, 32]. Dog owners are highly visible while walking the streets and in parks with their dog. Dog walkers may provide a daily source of informal surveillance; with more natural surveillance there is an increased likelihood of being observed or ‘caught in the act’ which may serve to discourage potential offenders [32]. Future research is required to confirm the observed associations between perceived neighborhood safety and dog walking status using large representative samples and context-specific measures of perceived safety associated with dog walking.

**Fig. 1:**
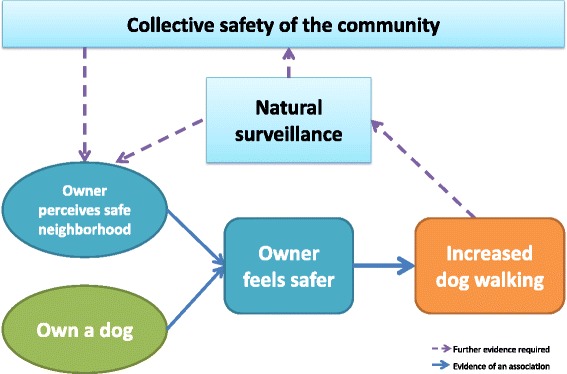
Theoretical model of the relationship between individual and community level safety and dog walking

Yet, we found no differences between dog walkers and non-dog walkers for how safe they felt in their neighborhood. However, when dog walkers were asked about how safe they felt when walking with their dog, significant gender differences emerged. Across all study sites a greater proportion of female than male dog walkers reported feeling safer when walking with their dog. This finding is consistent with a recent review of the correlates of dog walking which identified that perceived personal safety was positively associated with dog walking in females but not males [33]. Moreover, Suminski et al. reported that women were over three times more likely to walk their dog if neighborhood safety was perceived as average as opposed to below average [34]. It appears that there may be two ways in which safety could influence dog walking in females. First, simply having a dog to walk with could help women feel safer and second, a safer neighbourhood may support more dog walking. A proposed theoretical model of the relationships between dog walking and individual perceptions of safety and collective safety is shown in Fig. 1. The proposed relationships are based on the findings from the current study as well as published findings from our own and others studies [7, 9, 12, 16, 17, 33]. The proposed theoretical model highlights areas for further research to provide evidence of the relationship between dog walking and individual-level and collective safety.

This study observed a number of between city differences in dog walkers’ neighborhood perceptions. In particular, compared with dog walkers in Perth, dog walkers from each of the US study sites felt safer in their neighborhood, perceived that there was more neighborhood surveillance and perceived that they would feel safer if there was neighborhood surveillance. We also observed that a significantly higher proportion of Perth dog walkers (80 %) compared with dog walkers from the US study sites (range 28–45 %) walked in their local park. It is possible that if Perth dog walkers spend more time walking in their local park and less time walking the streets in their neighborhood, they may perceive lower overall levels of neighborhood safety and natural surveillance. These between city differences in dog walkers neighborhood perceptions may in part also be explained by social and cultural differences in dog-keeping (and exercising) practices as well as differences in the local physical and policy environment surrounding walking dogs in public [9, 35]. For example, there are significant differences between Australia and the US with respect to the design and access to public places for walking dogs. In the US, designated fenced dog exercise spaces are more common while in Australia dogs are more often exercised at large multi-use, multi-purpose unfenced urban parks or along footpaths on route to the local park [8, 13, 33, 36]. Further research is required to understand the social and cultural influences on the interaction between dog walking behavior and the neighborhood environment.

This study was limited by its cross-sectional design and thus causal relationships between dog walking, physical activity and owner’s perceptions of safety cannot be inferred. However, it involved a large representative sample from four cities in the US and Australia and provided a unique opportunity to investigate between and within country differences in a relatively new area of enquiry. This study was also limited by its use of self-report measures and did not examine gender differences in the relationship between dog walking status and neighborhood-level perceptions of safety. Moreover, the internal consistency (as measure by Cronbach’s α) for the scales measuring ‘Natural Surveillance’ were questionable thus, is it possible that these items measure unique aspects of natural surveillance related to safety. Future studies should explore the relationship between different constructs of natural surveillance and the relationship with dog walking and owner’s perceived safety.

## Conclusions

This multi-site international study provides further support of the potential for dog walking to increase the proportion of the community who engage in daily physical activity. Innovative strategies (e.g., promotion of dog walking) to increase physical activity levels are needed in developed countries where the burden of disease from insufficient adult physical activity is large [37]. Individual and community safety benefits associated with dog walking were also apparent. Among dog walkers, women were more likely than men to feel safer walking with their dog in their neighborhood. Walking with a dog may be a mechanism for getting to know the neighborhood and for improving levels of neighborhood natural surveillance and owner’s perceptions of safety. Significant between country differences were observed highlighting the need for further international research on the social and cultural influences on the relationship between dog walking and the neighborhood environment. This appears to be one of the first studies to investigate the relationship between dog walking and perceptions of neighborhood safety. Further studies in this emerging area of research may provide organisations and government departments responsible for providing community safety-based programs and policies the evidence required to support the wider community benefits of dog walking and strategies for increasing dog walking levels.

## Additional file

Additional file 1: Table S1. Adjusted within and between city differences in physical activity behavior by dog walking status. (DOCX 14 kb)
