# Supplementary material for: The association between dog walking, physical activity and owner’s perceptions of safety: cross-sectional evidence from the US and Australia
Source: BMC Public Health. 2016 Sep 22;16:1010. doi: 10.1186/s12889-016-3659-8 (PMC5034524; doi:10.1186/s12889-016-3659-8)
Supplement: Additional file 1: Table S1. — Adjusted within and between city differences in physical activity behavior by dog walking status. (DOCX 14 kb) [file 12889_2016_3659_MOESM1_ESM.docx]

Supplement Table 1. Adjusted within and between city differences in physical activity behavior by dog walking status

|  | San Diego  (n=276) | Portland  (n=233) | Nashville  (n=296) | Perth  (n=308) |
| --- | --- | --- | --- | --- |
|  | β (95% CI)^1^ | β (95% CI)^1^ | β (95% CI)^1^ | β (95% CI)^1^ |
|  |  |  |  |  |
| ≥30mins moderate-vigorous physical activity (days/week) | **0.73 (0.16, 1.29)*** | **0.82 (0.20, 1.45)*** | **1.17 (0.64, 1.71)***** | **0.92 (0.29, 1.55)**** |
|  |  |  |  |  |
| Frequency of neighborhood walking/week | **5.33 (4.51,6.15)***** | **4.20 (3.36,5.05)***** | **4.68 (3.87,5.48)***** | **4.30 (3.47, 5.15)***** |
|  |  |  |  |  |
|  |  |  |  |  |
|  | OR(95% CI)^1^ | OR(95% CI)^1^ | OR(95% CI)^1^ | OR(95% CI)^1^ |
|  |  |  |  |  |
| Walk in local park | **5.14 (2.61,10.14)***** | **5.84 (2.70-12.64)***** | **3.32 (1.61, 6.86)**** | **24.16 (11.50,50.75)***** |
|  |  |  |  |  |

*p≤0.05; **p≤0.01; ***p≤0.001; SD=San Diego; PL=Portland; NV=Nashville; PE=Perth

^1^ All models adjusted for age group, sex, highest education level, ethnicity (US); country of birth (Aust), number of children in household, housing type, time lived in neighbourhood; Reference group = Non-dog walker

^2^ Reference group = Perth
